# Supplementary figures and images for: Testing non-autonomous antimalarial gene drive effectors using self-eliminating drivers in the African mosquito vector Anopheles gambiae
Source: PLoS Genet. 2022 Jun 2;18(6):e1010244. doi: 10.1371/journal.pgen.1010244 (PMC9197043; doi:10.1371/journal.pgen.1010244)

Fig. S1

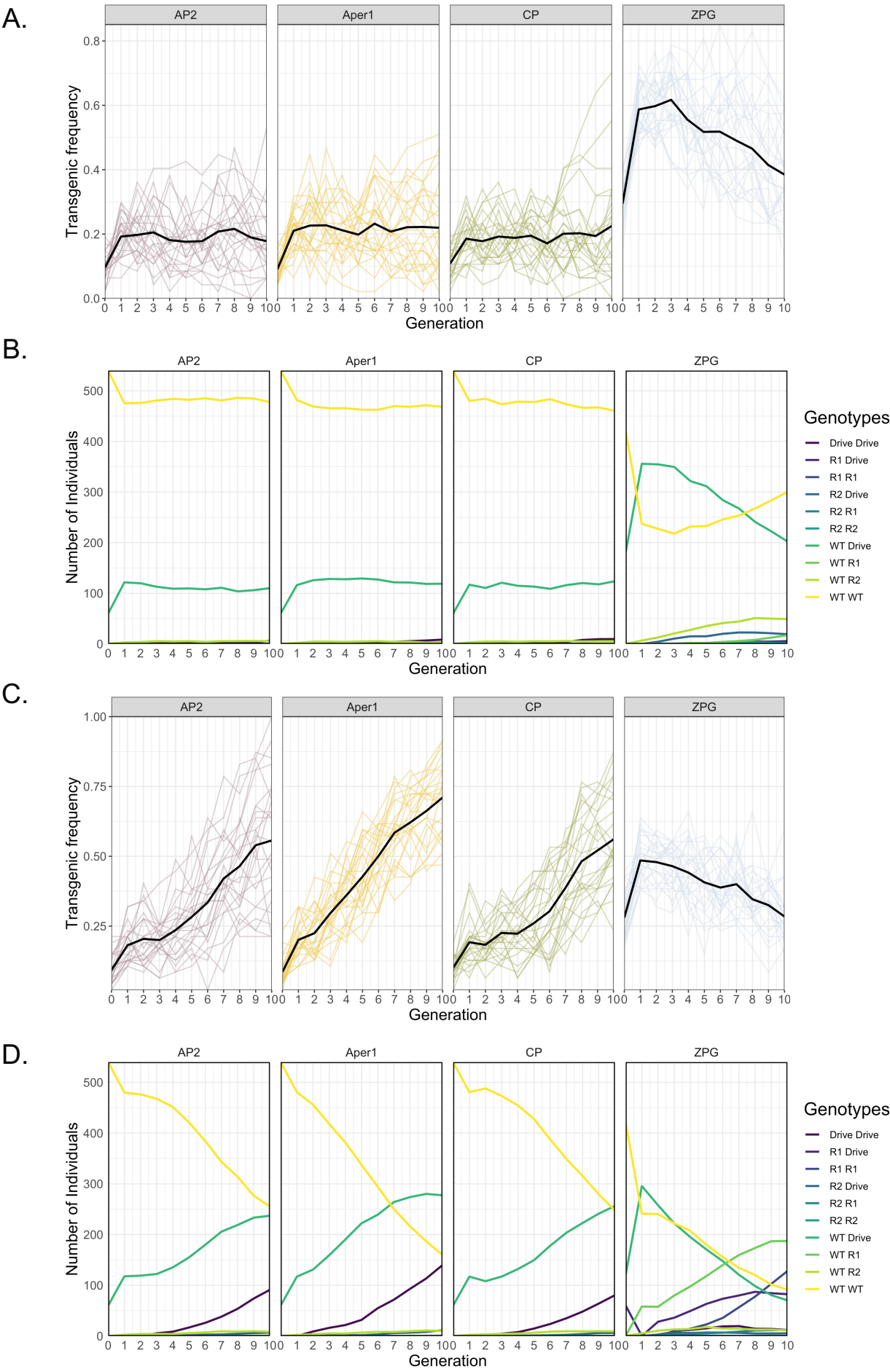

Supplement: S1 Fig — A: Stochastic simulation of populations of 600 adult mosquitoes with a starting population of 300 wild-type females, 120 wild-type males (all zpgS) and 60 zpgD/zpgS, Sco-CP/+ males; 60 zpgD/zpgS, ScoG-AP2/+ males and 60 zpgD/zpgS, Aper1-Sco/+ males. Shown are the average transgene frequencies at each locus calculated from 47 randomly sampled individuals out of 25 simulated populations. Because of this sampling strategy, which mimics our experimental one, starting transgene frequencies of zpgD (30%) and each of the payloads (10%) in individual populations may differ from the mean across populations (black line). B: The number of individuals of each indicated genotype at the 4 loci of the simulations in A of all 600 individuals in each population. Drive here refers to both autonomous (zpgD) and non-autonomous (Sco-CP, Aper1-Sco, ScoG-AP2) elements. C: Simulations as in A but assuming the presence of pre-existing R1 target site resistance at the zpg locus. These were introduced as 60 zpgD/zpgR, Aper1-Sco/+ males resulting in 5% starting allele frequency of zpgR. Shown are the average transgene frequencies at each locus calculated from 47 randomly sampled individuals out of 25 simulated populations. Because of this sampling strategy, which mimics our experimental one, starting transgene frequencies of zpgD (30%) and each of the payloads (10%) in individual populations may differ from the mean across populations (black line). D: The number of individuals of each indicated genotype at the 4 loci of the simulations in C of all 600 individuals in each population. Drive here refers to both autonomous (zpgD) and non-autonomous (Sco-CP, Aper1-Sco, ScoG-AP2) elements. (PDF) [file pgen.1010244.s001.pdf]

**Fig. S2**

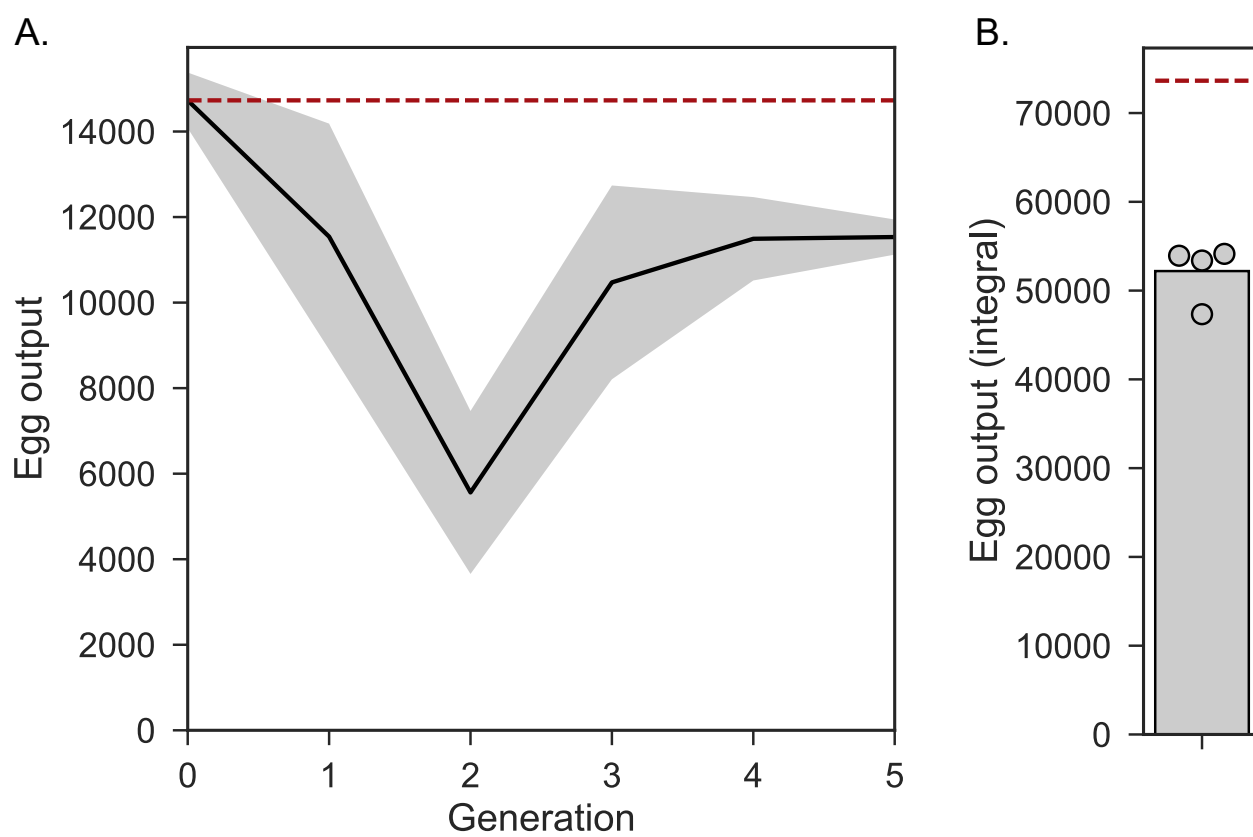

Supplement: S2 Fig — A: Total egg output of each caged population, at each generation shown in light grey; mean of all cages at each generation shown in black; predicted trajectory given no change in reproductive output (mean of all cages at G0) shown by dashed red line. B: The integral of each trajectory (grey circles) and their mean (grey bar) compared to the integral of the no-change trajectory (red dashed line). A one-sample t-test was used to compare the integrals of each real timeseries to that of the predicted no-change timeseries (t = -13.2, p<0.001). (PDF) [file pgen.1010244.s002.pdf]
