# Supplementary material for: Testing non-autonomous antimalarial gene drive effectors using self-eliminating drivers in the African mosquito vector Anopheles gambiae
Source: PLoS Genet. 2022 Jun 2;18(6):e1010244. doi: 10.1371/journal.pgen.1010244 (PMC9197043; doi:10.1371/journal.pgen.1010244)
Supplement: S1 Methods — (DOCX) [file pgen.1010244.s006.docx]

**Gene Drive Modelling using SMS**

We employed a simple object-oriented stochastic agent-based discrete-generation model written in C# which we termed SuperMendelianSandbox (SMS). The model is written for gene drive prototyping and emphasizes human readability of the code which, together with the specific simulation parameters used in this study, has been made available here: <https://github.com/genome-traffic/SuperMendelianSandbox/tree/zpg_lim>. The model allows to fully account for the genetic interactions of autonomous and non-autonomous CRISPR-based drive elements as well as the generation and tracking of R1 (functional) and R2 (non-functional) resistance alleles separately at all modelled loci. The model’s hierarchical structure features at the lowest level the *GeneLocus* class that describes individual genomic locations and tracks the gene and allele names (WT, R1, R2 or Transgene), position on the chromosome and the *Traits* list of locally encoded traits or properties. Traits include the functional conservation of a gene (the probability that R1 or R2 alleles are being generated), the local rate of homologous repair or the levels of Cas9 or of the gRNAs encoded within the gene locus. It includes methods to determine genetic distances and recombination frequencies between loci (*RecFreq, Distance*) and to compare *GeneLocus* objects for identity (e.g. *IsSameAllele*). The *Chromosome* class describes chromosome objects that feature a list of *GeneLocus* objects and provides various *Chromsome* constructors. The two most important constructors take as inputs two existing homologous chromosomes and, analogous to meiosis, resolve cleavage and homing events via the *CutAndHomeInto* method as well as recombination events based on genetic distance to generate an output chromosome. The *Organism* class describes organism objects that feature two *Chromosome* lists for the two sets of homologous chromosomes and tracks maternally (or paternally) deposited factors (e.g. Cas9 or gRNAs listed in *ParentalFactors*). Organism constructors include the ability to generate a child organism by providing a male and female parent organism and resolving all chromosomes via meiosis by the *GetGametChromosomeList* method. The *Organism* class also provides organism-level methods to determine zygosity of particular genes and transgenes (e.g. *AlleleHomozygous*, *AlleleHeterozygous* methods), determine the organism’s sex (*GetSex*, *IsMale*, *IsFemale* methods) or fertility (depending on the genotype of each of the tracked genes as defined in the *GetFertility* method) or to calculate the level of certain gene products by summing across various expressing loci (e.g. Cas9 being provided by multiple transgenes on different chromosomes) via the *GetTransgeneLevel* method. The *Population* class hosts the global list of adults and eggs and describes methods that define and return organisms of various types including basic constructors for wild-type populations. It also features the *PerformCross* method that allows to trigger reproduction of a mating pair. The *Simulation* class, in addition to allowing to define most global parameters (e.g. establishing a match between target genes and cognate gRNAs, the population cap or the standard female fertility), provides the main method *Simulate* which resolves the random mating within a population to establish the following generation and is also responsible for all data output. Each generation all tracked genes (as defined by the *Track* array) and their genotypes as well as basic population statistics (the number of eggs laid, male and female individuals, etc.) are output as comma separated value format. R scripts to analyse SMS output files are available under <https://github.com/genome-traffic/gRandTheftAutosome>. For simulating the cage population dynamics of zpg^D^ in conjunction with the 3 payload loci we considered populations of 600 individuals and independently segregating 4 genetic loci where WT, R1, R2 and Drive constitute the possible genotypes at each locus. We included the experimentally observed sex-specific fertility defects of zpg^D^ alleles and fitness costs for loss of zpg. This includes a complete loss of fertility for females with any combination of zpg^D^ and R2 alleles and for zpg^D^/zpg^S^. In zpg^D^/zpg^S^ males we assumed a 59% drop in fertility. The main assumption in the model was the fitness cost of loss of function at the 3 payload loci where we lack experimental data. Here, R2/R2 allele combinations at any of the 3 payload loci were accumulatively accounted for as a 25% drop in fertility. We assumed near optimal Cas9 activity (99%) and a rate of homologous repair of (96%) at all loci with R1 alleles being generated at a rate of 5% at all loci.
