# Supplementary material for: Testing non-autonomous antimalarial gene drive effectors using self-eliminating drivers in the African mosquito vector Anopheles gambiae
Source: PLoS Genet. 2022 Jun 2;18(6):e1010244. doi: 10.1371/journal.pgen.1010244 (PMC9197043; doi:10.1371/journal.pgen.1010244)
Supplement: Attachment — Submitted filename: Ellis et al PLOS Genetics 3-28-2022.docx [file pgen.1010244.s008.docx]

My Review of: “**Testing non-autonomous antimalarial gene drive effectors using self-eliminating drivers in the African mosquito vector *Anopheles gambiae”*** by: David A. Ellis1,2, George Avraam1, Astrid Hoermann1, Claudia A. S. Wyer1,4, Yi Ong1, George Christophides1, Nikolai Windbichler1,*

This manuscript describes an important advancement to Integral Gene Drives, developed in mosquitoes previously by Nash et al. in 2019, in which the drive element (Cas9-expression) is integrated into an essential fertility gene in such a way as to incur a high fitness load and so be selected against, ensuring its depletion from a population overtime, while simultaneously effecting introgression of separate, non-drive, antipathogen transgenes into cage populations by the drive element. Specifically, the zpgD transgenic allele is shown to drive three different effector modules, while itself decreasing in frequency over generations. This is an important development in mosquitoes with multiple merits for the potential of confineable drives, but the greatest I see is in how useful it will be for testing multiple transgene arrangements, simultaneously at a larger scale. The focus on minimal design elements improves the potential fitness loads associated with any given transgene. These are of high importance to our field.

The authors provide a thorough genetic analysis of the activity of their transgenes at the molecular level, in genetic crosses and cage populations. Beside providing compelling evidence for their claims, they demonstrate that genetic/molecular phenomena must be carefully examined in genetic crosses and cage populations because different guideRNAs, effectors, insertion locations and expression strategies, can have very different effects.

The authors detail a thorough genetic analyses demonstrate technical molecular phenomena:

-Variation in the target site in zpgR individuals confers resistance to the drive which is demonstrated to be important to the drive dynamics. This set of experiments highlight the need for accurate documentation of alleles in the target population to predicting and understanding movement of the transgene through the population.

-guideRNAs very close together at the target drive the transgene differently 65% compared to ~95% in figure 2A, which is important in a drive.

-Readthrough translation is indeed inefficient in this system, which was useful to this design.

This manuscript is well written throughout. The visuals in the figure are effective at illustrating the complex genetics and the implications of the data. There is a nice review of effector genes so far available in the introduction.

Minor improvements to be made:

The terms “homing-induced homozygosity,” “gene-conversion” and others are useful in understanding what is happening with the drive in the germline, which isn’t intuitive to scientists outside the field, but the first term isn’t used until the discussion. Could you use some of this language earlier in the paper?

It is essential for reproducibility of these strategies and for application to other mosquito systems for annotated constructs (particularly, pZPGCas9Dr_v2_ZPG1 and pZPGCas9Dr_v2_ZPG2) to be made publicly available, such as on GenBank upon publication.

Some improvements to the discussion should be made:

The authors claim that there is an advantage in using a drive that is integrated in such a way as to be selected against. In this case integration of the Cas9- and marker-expressing cassette into the open reading from of zpg reduces the proper expression of this gene. Can the authors discuss existing data on drive elimination in a “neutral” location for perspective on this advantage?

None of the non-autonomous elements reached fixation in the cage population (or maybe 1 did-ScoG-AP2). With decreasing Cas9 in the population, would we expect to see fixation of any of the non-autonomous elements? Perhaps in many of the cages, by generation 6, the allele frequencies were high enough we might expect them to continue to increase, or would the begin to decrease from a fitness load?

With the reduction in egg laying demonstrated, does this strategy resemble a “reduce and replace” strategy?
