# Supplementary material for: Testing non-autonomous antimalarial gene drive effectors using self-eliminating drivers in the African mosquito vector Anopheles gambiae
Source: PLoS Genet. 2022 Jun 2;18(6):e1010244. doi: 10.1371/journal.pgen.1010244 (PMC9197043; doi:10.1371/journal.pgen.1010244)
Supplement: Attachment — Submitted filename: Response to Reviewers 1.3.docx [file pgen.1010244.s009.docx]

Recommended Additional Revisions (Editor):

The authors assume that zpg is required in germline cells, so drive/wild-type individuals become sterile by germline drive conversion. More likely than not, this is correct, but it dismisses another possibility that could explain all or some of the sterility. Based on previous studies of zpg-Cas9 in suppression drives, it is known that zpg leads to some “leaky somatic” expression in somatic cells, which moderately reduces the fertility of females (by cutting the wild-type allele). It might be that this is an important factor in reducing zpg expression in important cells, so the possibility should at least be mentioned.

***Reply****: This is an important possibility which we now account for. On page 7, we added text highlighting this point, referencing a previously cited paper where the promoter was extensively characterised. New text (underlined): “However, homozygosity of zpg^D^, and probably homing-induced homozygosity in the germline of hemizygote females, led to fertility defects. Note that some low level somatic “leakiness” has been reported when using the zpg promoter for germ-line expression [22]**, and hemizygote infertility could also result from expression of zpg^D^ in the soma.”. We also clarify this further down on page 8: “Because the active homing of zpg^D^, or its leaky somatic expression, results in female sterility…”*

For the discussion about Figure 5, I think it might be helpful to take a step back and introduce for a more general audience what makes a functional resistance allele and why seeing these at high frequency is a pretty good reason to assume that they are r1 (compared to r2). It also seems a little odd to me that more possible r2s were not detected in G1 (compared to G6). Maybe there was a mechanism to quickly eliminate them.

***Reply****: Regarding the discussion of functional resistance, we have added a comprehensive introduction of resistance allele generation and functional resistance for a more general audience. All citations were in the previous version of the manuscript. Instead of the discussion, we have put this before going into the results for clarity. New text (underlined):*

*“Following cleavage by Cas9 in the germline, DNA double-strand breaks can be resolved by the DNA repair machinery in several ways. Homology directed repair (HDR) using the drive allele as a repair template leads to conversion of a wild-type allele to a drive allele (i.e. homing). Due to the design of gene drives, this outcome eliminates the gRNA target site making the new drive allele resistant to future cutting. However, DSB can also be repaired via DNA end-joining pathways such as non-homologous end joining (NHEJ) or microhomology-mediated end joining (MMEJ) which does not lead to drive conversion. The resulting small deletions or insertions generally destroy the gRNA target site and abolish recognition by Cas9. Such cleavage resistant alleles, which can also occur naturally as standing genetic variation of a population (as is the case for zpg^R^), present a barrier to gene drive. These mutations may also arise outside of the germline in developing embryos as a result of maternally deposited Cas9 [19,20,22,41]. With respect to target gene function, the impact of such mutations can range from being detrimental (termed R2 alleles) to being neutral, for example when they result in synonymous substitutions or small tolerable deletions that maintain the reading frame (termed R1 alleles). R1 alleles that present a lower relative cost than a circulating drive will therefore undergo strong selection [19,41,42,52,53].”*

*Given the new extensive introduction of the concept, we have removed a sentence in the original paragraph that previously referred to R1 and R2 alleles. Furthermore, having now defined non-homologous and microhomology-mediated end-joining, we refer to them as NHEJ and MMEJ further down in the text. A previous reference to homology directed repair in the methods has also now been abbreviated for similar reasons.*

*Regarding the low abundance of R2 alleles at G1, this may simply reflect the fact that in G0 homing can only occur in males. (This is also the reason why zpg^D^ increases in frequency from G0 to G1)*

The modeling section in the methods should be considerably expanded. Including the code is good, but the methods should still fully describe the model. If needed, it can be a supplemental methods section.

***Reply****: We have provided a supplemental methods section describing the components of the model in greater detail.*

Minor Comments and Suggestions (revisions at the discretion of the authors):

I liked reviewer 2’s comment on population replacement versus population modification. I agree with the terminology myself as well.

***Reply****: We are now using the term “population modification” throughout.*

Please take care to include line numbers or at least page numbers when submitting to journals. Some add it automatically, but this copy was not marked with line or even page numbers.

***Reply****: Page and line numbers have been added to our revised manuscript.*

Author affiliation #3 does not seem to be used.

***Reply****: This has been rectified in our revised manuscript.*

Split drives are reference in the introduction [23,24,26,32,33], but this actually underestimates how widely they are used for biosafety or other reasons. Here are some more (this might even be missing some):
https://elifesciences.org/articles/41439 (first split drives in insects)
https://www.liebertpub.com/doi/10.1089/crispr.2021.0129
https://www.pnas.org/doi/10.1073/pnas.2004373117
https://www.sciencedirect.com/science/article/pii/S2211124720308226
https://journals.plos.org/plosbiology/article?id=10.1371/journal.pbio.3001478 (I think the other two mouse papers also used split drives)
https://www.nature.com/articles/s41467-020-14960-3

***Reply****: Although the original references were not meant to be comprehensive, we have bolstered them with those mentioned above, including the further two mouse papers mentioned. New text (underlined): “[23–26,32–40]”. One of these eight references is already cited elsewhere in the manuscript (doi.org/10.1016/j.celrep.2020.107841).*

The long-term behavior of the cage populations would be interesting, such as where the final equilibrium values fall past generation ten. If the authors have kept the cages going, it would be nice to include this data in the manuscript (even just a few more generations), but this is certainly not necessary.

***Reply****: Unfortunately, this population trial had to be terminated by generation 6 due to COVID-19-related challenges. We would also have loved to see its progress beyond that which we show.*

**Reviewer #1:** In this paper, the authors present the testing of the direct integration of a Cas9 drive cassette at the zpg locus in Anopheles gambiae, with a novel readthrough strategy to minimise modifications. They go on to investigate the impacts of resistance alleles in a target population, analysing homing rates, fitness consequences and drive dynamics to test the efficacy of a system as a proposed non-autonomous drive.
The paper is technically very interesting, well written and clear throughout, and potentially of significant importance in the continued development and refining of gene drive systems for pest control.
I summarise a list of primarily minor comments that I hope will improve the manuscript for publication.

Main comments:

I have some concerns about data management that I hope are easily fixable by the authors. I cannot find the raw data to accompany fertility assays and cage trials; I also cannot see a link to a data repository. Supplementary Table 1 appears to be infection data, the analysis of which I cannot see mentioned anywhere in the manuscript.

***Reply****: Supplementary tables for the fertility assays and cage trials are now provided.*

*The original draft of the paper contained infection assays using Plasmodium berghei. We confirmed that zpg^D^ induced female sterility and ovary atrophy led to lower infection levels as suggested by the literature. However, as we had only a single replicate and the data didn’t fit with the rest of the story this was removed. If there is strong interest this data could be added back. For now, we have removed Supplementary Table 1.*

The authors summarise one of the uses of this drive as "Self-eliminating drivers could thus enable local population modification without leaving behind any driver element.” However – in this scenario the zpg drive has a suppressive effect on the population. As effector frequency rises within a reducing effective population size, the effect of migration into the release area will change the drive’s dynamics. I would like to see the authors discuss the potential implications of migration even if it is not formally incorporated into any models.

***Reply****: Thank you for this very interesting insight. We thought this discussion fit well with reviewer 3’s final comment on the parallels between the system presented here and ‘reduce and replace’ systems. Using this literature, we have tried to write a reasonably comprehensive summary of the possible implications of migration on zpg^D^-based population modification. We have also tried to include some population genetic aspects of the drive that we did not cover and require future examination.*

*New text (underlined): “Multigenerational trials using caged populations can tell us a great deal about a gene drive system, but there are fundamental aspects of field populations that cannot be recapitulated in a lab setting. Whilst our simple discrete-generation model goes some way to predicting the behaviour of zpg^D^ and interdependent transgenes, important aspects of population genetics could not be explored or have not been explicitly investigated. For example, several features accompanying a possible zpg^D^ release could lead to a reduction in the effective population size. These include the strong selection for resistance alleles, the imbalances in reproductive output between transgenic males and females, or between transgenic and wild-type individuals, and the reduction in census population size due to zpg^D^’s suppressive effect. This last point is particularly important as it also affects migration (which, in-turn, can influence the effective population size). For example, the smaller a target population is relative to a neighbouring population, the more immigrants it will receive per emigrant, with immigrants also making up a larger proportion of the population [60]. Such an effect is evident in stochastic, spatially-explicit modelling of so-called “reduce and replace” systems [61], where population size is reduced by a female-specific lethal transgene whilst an anti-pathogen payload is transmitted through surviving transgenic males [61,62]. It has been suggested that, with a greater level of population suppression, the effect of wild-type migrants on transgene dynamics is amplified, impacting the long-term frequency of the payload gene. Parallels between this approach and zpg^D^-based population modification would suggest that we may find similar effects in the early stages of a release where zpg^D^ is at a high frequency and the population is being suppressed. On the other hand, this asymmetry could be beneficial for migration in the other direction i.e. reduced escape of transgenes and invasion of non-target populations (although note that others have suggested that in many cases, escape is still possible with relatively low levels of migration [63]). Lastly, any effects of, or from, migration will depend strongly on the frequency of resistant alleles in neighbouring populations [64]. Future work will need to determine the importance of these factors in influencing the dynamics of released gene drives.” This text contains 5 newly added citations.*

Minor comments

Transmission of zpgD and a payload in susceptible and resistant genetic backgrounds:
The authors introduce the Sco (Scorpine)-CP(Carboxypeptidase) line, this is used in a nice experiment to demonstrate that lack of homing of the zpg cassette is due to resistance at that locus and not lack of Cas9 activity; but while it is referenced in another paper it would be nice if the authors could briefly expand into the full names of these constructs and loci here, especially as they mention Scorpine in full elsewhere.

***Reply****: We have improved the description of the line at the point you identify. New text (underlined): “...we generated transhemizygous females carrying zpg^D^ in combination with a zpg^R^ allele, as well as the previously characterized non-autonomous payload drive Sco^GFP^-CP, which harbours a gRNA targeting the CP wild-type allele but lacks Cas9 [43]. This non-autonomous drive element expresses the antimicrobial peptide Scorpine [51] from within the CP host gene which is strongly induced upon bloodfeeding. When crossed to wild-type males in single mating pairs we found that, as observed previously, zpgD was transmitted in Mendelian fashion to the progeny. In contrast, ScoGFP-CP was transmitted to nearly all progeny...” This text contains a reference already cited elsewhere in the manuscript.*

Fertility and gonad development of zpgD transgenics:
In this section the authors make multiple comparisons between homozygous/heterozygous males and females. The experiments are clear, but some aspects of the analysis need clarity or could be improved. There are many pairwise comparisons here
with t-tests (Student's or Welch's? this is undefined), and it is not clearly explained whether these are first tested across one general linear model before diving into these pairwise comparisons. Some of the distributions of data (in some groups there are no offspring) seem ill-suited to a t-test, but I also don't think incorrect conclusions have been drawn here because of the large effect. Although some sample sizes are given at the beginning, it is important to include the degrees of freedom for each t-test as this will determine the critical threshold for statistical significance.

***Reply****: Regarding the excess of pairwise comparisons performed, we have now limited the statistical tests to only those groups which make sense, as per your comment below regarding the comparison of males offspring number with females. Regarding comparisons to groups with no data in Figure 3B, the statistical comparison of hemizygous females to homozygous females (where no offspring were produced) has now also been omitted, and we simply refer to the stark difference shown by the data (as per our response to reviewer 2’s 15th comment). The only test in this analysis is now between hemizygous and homozygous males, both of which have non-zero data, where we use a Welch’s t-test due to the unequal sample sizes. This is now referred to in the text (see reply to reviewer 2’s 16th comment). Regarding the degrees of freedom, we have now report them in brackets next to each test.*

The comparison between male and female transgenics and offspring production does not seem like a 'fair test' I would be surprised if even with sperm depleted males, they could not fertilise all wild-type female eggs - a more thorough comparison would compare them in sperm competition with wild-type males.

***Reply.*** *A fair point, we now only compare within the two groups.*

When discussing gonad morphology, the measure for ovary atrophy is not described.

***Reply.*** *We have added a sentence to the results section to clarify. New or altered text (underlined): “Pronounced atrophy of the ovaries was observed in both zpgD/zpgS and zpgD/zpgD females (Figure 3C). This was also quantified using measurements along the major axis (anterior-posterior) of each ovary (Figure 3D; t=8.342, p<0.0001, and t=7.988, p<0.0001, respectively; independent two-sample t-tests comparing each genotype to wild-type).”*

Population cage dynamics of zpgD in combination with payload drives:
The authors describe the drive of a non-autonomous element in the presence of target-site resistance at zpg as seemingly paradoxical. But I'm not sure why they describe this as such, many split-drive designs have previously been discussed, this seems perfectly logical.

***Reply.*** *We have removed “seemingly paradoxical” from the sentence.*

When describing the " introduction of resistant alleles would induce gene drive and increase effector frequency at the 3 payload loci" - could the authors summarise this effect?

***Reply.*** *We now briefly summarize the reason for this. New text (underlined): “...we found that the introduction of resistant alleles would induce gene drive and increase effector frequency at the 3 payload loci (Supplementary Figure 1C,D). This was due to active drive of the payloads now also occurring in zpg^D^/zpg^R^ females, which unlike zpg^D^/zpg^S^ females, were completely fertile.”*

Figures
Figure 1B - label that the grey letters indicate SNPs

***Reply****. This was now indicated in the legend. New text: “Nucleotides highlighted in grey are SNPs.”*

Figure 1D - the scale and colours are not the easiest to read.

***Reply****. Agreed, although other colour schemes didn’t seem to improve this. The basic point that the SNPs of the S allele predominate in some populations but not in others is hopefully coming across.*

**Reviewer #2:** In an effort to enrich the toolbox for gene drive in mosquito vectors of human malaria, the authors sought to establish a driving Cas9 transgene integrated non-disruptively within the coding sequence of the essential zpg mosquito gene, to serve as a Cas9 source for spreading some non-autonomously driving antimalarial transgenes in a target mosquito population. Somewhat unexpectedly, Cas9 insertion at zpg had a large fitness cost as it impaired zpg activity, especially in females, resulting in strong selection for pre-existing zpg alleles resistant to Cas9 cleavage, counteracting the drive. The authors turn this defect into an advantage as it provides a system for transiently sustaining Cas9 in a mosquito population and reach some level of spread of a set of 3 non-autonomous, gRNA-encoding transgenes, before inexorable disappearance of Cas9 due to the rise of non-costly resistant zpg alleles.
I agree with the conclusions of this study and with most discussion points. The concept and experimental demonstration of a full Cas9 gene drive that self-eliminates after some period of activity, on its own and on distant loci, is quite novel (the molecular mechanism is different and more simple than for the previously theorized “daisy drive” strategy). Programmed disappearance of Cas9 could be partially reassuring to some critics of the gene drive technology, even if the driven non-autonomous transgenes (technologically less frightening) could persist. I appreciate the model of malaria control by gene drive mosquitoes using a combination therapy-like approach through multiple independent anti-malarial transgenes, all driven by the same Cas9 locus. The experimental procedures employed to obtain the results (genotyping and combining defined proportion of mosquitoes of desired genotypes in genetic crosses and follow-up of the frequency of 4 transgenes by genotyping 47 individuals over 6 generations) represent a considerable amount of work.

This work will be of interest to the gene drive field (a contemporary hot topic) and to evolutionary geneticists. It will also be read by stakeholders in mosquito control, including the risk assessment community, and more generally by people interested in and/or worried by gene drive and biotechnology at large. I would be happy to see this study published, once the following points have been addressed or discussed:


Introduction: in an effort towards clarifying vocabulary in the gene drive field, I suggest to reserve the term population replacement for gene drives based on underdominance (whereby the genomic background of the released strain should entirely overwrite that of field populations); and population modification for gene drives that introgress novel genetic traits into a field population’s genetic background without altering the bulk of it (as would be the case with the approach presented here). It’s up to the authors to adopt this point of view or not, if so using “population modification” throughout the manuscript.

***Reply****: thank you for your suggestion. We will adopt this naming system for the paper - see our reply to the editor’s first minor comment.*

Introduction third §: I believe the authors mean “to the expense of the drive” instead of “in favour of the drive”.

***Reply****: Correct, this was rectified.*

A bit further: note that if a non-driving Cas9 insertion is costly, it should still disappear by natural selection.

***Reply****: We now state this explicitly. New text (underlined): “However, this does not apply to non-driving Cas9 transgenes which cannot trigger their own replacement by resistant alleles (although note that if costly, they should still disappear by natural selection).”*

Last § of intro: for more clarity write “capable of providing the antimalarial transgenes with a source of Cas9”….

***Reply****: This adjustment was made.*

Results: construct design is not immediately clear: it may be useful to mention that the antisense 3xP3-Scarlet reporter gene must be devoid of any stop codon to allow readthrough translation (also in fig 1B legend). In fact I wanted to check for the absence of stop codons in the antisense 2A-3xP3-Scarlet-2A cassette but the nucleotide sequence of the final construct is not provided. Given the complexity of the cloning scheme used to obtain it, providing an annotated sequence file of the whole construct (plasmid or integrated in its genomic context) as a Suppl. file is required. What might be the physiological effect of expressing the nonsense peptide arising from antisense Scarlet and 3xP3 in zpg-expressing cells? It could contribute to the fitness cost.

***Reply****:We now provide the plasmid files used to generate both transgenic lines in this study (pZPGCas9Dr_v2_ZPG1 and pZPGCas9Dr_v2_ZPG2) as a supplementary file. There are no stop codons in the antisense Scarlet marker and regulatory elements. Regions that were recoded to achieve this are annotated.*

*As for the effect of the nonsense peptide on germline cells, this is unknown. We now mention the possibility that it may contribute to the fitness cost. New text is described along with other additions in response to your 20^th^ comment.*

Result § “transmission of zpgD…”: for clarity add: “to determine their zpg R or S status, hemizygous parents were then genotyped…”

***Reply****: This clarification was added to the text.*

To guide the reader could also add: “Figure 2A, purple bars / Figure 2A, yellow bars”

***Reply****: The colour of the bars is now mentioned when refering to each result. New text (underlined): “...we found Mendelian inheritance of the zpg^D^ allele when it was paired with the zpg^R^ allele... (Figure 2A, purple bars)… When, in transgenic males, the zpgD allele was paired with the susceptible zpgS allele... (Figure 2A, orange bars).”*

Fig. 1C: I’m not convinced of the necessity of the left panels with chromatograms, redundant with Fig 1B. Fig. 1D: why not indicate the polymorphic nucleotides and their position in each strain, not only G3. The heat map for allele frequency is visually not very clear (consider blue to red rather than white to red, to make the dynamic range of frequencies more contrasted?)

***Reply****. The chromatograms are meant as evidence of the genotype at the PCR products used for cleavage experiments. Regarding 1D, we did not feel different colour schemes improved the basic point of the graph that some SNPs from the S allele predominate in some wild populations but not others (see response to reviewer 1), however we now indicate the nucleotides throughout.*

Fig. 1D I think the correct spelling of the strain name is Ngousso, a suburb of Yaoundé in Cameroon. Also in Materials & Methods.

***Reply****: This was fixed in text and figures.*

Fig 3A The purpose of showing PCR products 2a and 3 is not obvious here (they’re not on the associated DNA gel), I didn’t notice an explicit reference to them in the text.

***Reply****: These primer sets were used for large scale screening as described in the figure legend and methods. We have added text when referring to the figure to bring clarity. New text (underlined): “First, using primers annealing to genomic regions external to the homology arms of the template plasmid, we performed long-range PCR on individuals from this pool (Figure 3A, primer set 1 and gel image)… We used PCRs to genotype individuals, and sequenced zpg alleles in those that were hemizygous (Figure 3A, primer sets 2 and 3; see methods).”*

B of figure 3B is missing.

***Reply****: This was fixed.*

Text referring to figure 3B: speaking of insemination rate suggests that spermathecae were dissected and counted to assess the number of females that received sperm, independently of sperm fertility. I see in the Methods that this was indeed done but here the number of males that successfully generated progeny is discussed => replace “insemination” by “male reproductive success / ability” ?

***Reply****: This section was generally unclear so we have reworded it. New text (underlined): “To better understand the effects of different allelic combinations at the zpg locus on male and female fertility we measured life history and physiological traits in a set of individuals from this pool. In single pairs, we crossed individuals to the wild-type and recorded: 1) whether the copulation was successful (based on whether or not the female laid viable eggs, and if they didn’t, the status of their dissected spermatheca), and 2) the number of offspring that were sired. 46 male crosses and 46 female crosses were analysed in total. We used PCRs to genotype individuals, and sequenced zpg alleles in those that were hemizygous (Figure 3A, primer sets 2 and 3; see methods). The following were successfully genotyped: hemizygous females n=24; hemizygous males n=33; homozygous females n=17; homozygous males n=10. Of the 33 crosses with zpgD/zpgR fathers, 12 led to successful insemination of females (36%), whereas of the 10 crosses with zpgD/zpgD fathers, only 2 were successful (20%). RNA interference (RNAi)-mediated knock-down of zpg has been previously reported to lead to completely sperm-less males [52]. Whilst we found no significant difference between the insemination rate of hemizygous and homozygous zpgD males (X2=0.936, p>0.05), the low insemination rates, particularly of zpgD/zpgD males, suggest zpgD may display some phenotypic manifestation of reduced zpg expression. Next we analysed the number of offspring sired by each genotype. Whilst hemizygous (zpgD/zpgR) females produced an average of 79 ± 26 offspring (mean ± standard deviation) – a similar number to hemizygous males (74 ± 34) - from those homozygous (zpgD/zpgD) females that mated and laid eggs, none generated offspring (Figure 3B). This was not the case in homozygous males where viable progeny was produced (26 ± 18, mean ± standard deviation). Due to their unequal sample sizes, a Welch’s independent two-sample t-test was used to compare the number of offspring sired by the two male genotypes (Welch’s t(df=2)=3.035, p=0.070), although non-significance is probably due to the low number of homozygous males. Whilst there may be some impact on male fertility, viable progeny were still sired by both genotypes in males, suggesting that zpgD maintains some expression of functional zpg gene product in males.”*

In the parenthesis referring to Figure 3C,D you could precise “comparing gonad size in each genotype compared to the WT” for more clarity

***Reply****: We have hopefully improved the clarity of this section in response to reviewer 1’s 4^th^ minor comment.*

Suppl Fig 1A: could mention in the legend that all theoretical curves don’t start exactly at the initial G0 frequency of 0.15 for zpgD and 0.05 for each antimalarial transgene due to simulated random sampling (I was initially confused)

***Reply****: Correct, although the subsampling was only applied to panels A and C (to mimic the amount of data and readout we would get from our actual cages) which show transgene frequencies. We added the following text to the legends for A and C:* “Because of this sampling strategy, which mimics our experimental one, starting transgene frequencies of zpg^D^ (30%) and each of the payloads (10%) in individual populations may differ from the mean across populations (black line).”

Where 3B is cited, it’s a bit awkward to write that “none” is “significantly fewer” than (anything).

***Reply****: We now changed this to state: “This was not the case in homozygous males where viable progeny were produced (26 ± 18, mean ± standard deviation)”*

Non-significance of the number of progeny sired by zpgD/D compared to zpgD/R is certainly due to the very low sample size for D/D.

***Reply****: We now mention this in the text.*

*“…Due to their unequal sample sizes, a Welch’s independent two-sample t-test was used to compare the number of progeny sired by the two male genotypes. This was narrowly non-significant (Welch’s t(df=2)=3.035, p=0.070), although this is probably due to the low number of homozygous males.”*

End of the Results section: not immediately obvious what is meant by “mutations distal to the predicted cleavage site” → relatively distant from… Note that the last shown zpg mutant allele should not be a resistant allele, as the TGG PAM is simply changed to GGG, also a valid PAM. I wonder if those mutants with a very distal mutation (e.g. first one for CP) would truly block Cas9 cleavage. Do the authors think these distal mutations arose from Cas9 activity, or are pre-existing polymorphisms detected or not in the 1000 genome sequencing project? The more distal they are, the more likely they’re to be pre-existing polymorphisms. If so, such alleles should not be called mutations but SNPs. Is this what the authors wanted to note in the legend of figure 5B ("Note that for some loci (e.g. Aper1) many, low-frequency alternative alleles exist that are neither R alleles nor reference alleles") ? This note is not so clear and it’s hard to know whether a given allele is R or not. Actually, could these polymorphisms simply be PCR mutations that become visible in high-throughput amplicon sequencing?

***Reply****: Regarding the first point, we have changed the text to read: “mutations relatively distant from the predicted cleavage site”.*

*Regarding the TGG to GGG substitution, we have added a sentence to the methods section identifying that the (endonuclease agnostic) analysis does not discriminate these occasional mutations from the others. New text (underlined): “Using the frequency tables of alleles around the sgRNA produced by CRISPResso2, a custom Python script was used to select only those alleles with mutations in the region bound by the gRNA spacer or the PAM (i.e. those that would potentially cause resistance to gene drive).*

*Regarding whether distal mutations arose from Cas9 activity, or are pre-existing polymorphisms, it may be that those nearer the cut site are more likely to have been generated by Cas9 activity. However, because it is impossible to tell categorically, we have ignored this distinction.*

*Regarding the misleading note: we sequenced 2x300bp reads. In panel A we show the percentage of these reads that contain an R-allele (i.e. reads with a polymorphism in the gRNA + PAM). Although it is not important for the results of the analysis, it is possible that some readers may assume that the remainder of these reads must be reference, as they do not contain an R-allele. However, what CRISPRESSO categorises as ‘reference’ is precise (i.e. exactly the same as the reference sequence that was aligned to) and many of these reads will contain polymorphisms elsewhere in the read, meaning they are not categorised as ‘reference’, even though they are not R-alleles. This note was meant for readers’ whose assumed percentage of reads were at odds with those shown in part B. We see how it is misleading and have removed it.*

*Regarding PCR artefacts, we have not seen those when we did amplicon sequencing of WT samples for example. As can be seen in figure 5 similar variants come up in the experimental replicates thus ruling PCR errors out as a major source of variation. Furthermore, we would not expect a relative increase in the number of PCR errors with generation time. In this respect, each locus has its own baseline control (i.e. G1).*

It’s actually quite amazing that for Aper and AP2 the resistant alleles gaining most frequency at G6 are very distal mutations that look like they should not completely block Cas9 cleavage. This may suggest that even single very distal mismatches efficiently preclude cutting in Anopheles.

***Reply****: In both cases the mismatch is at position 16 of the gRNA target so not sure that our data allows us to draw such a strong conclusion.*

Discussion: please rephrase “and the zpgD Cas9 module was linked with the endogenous zpg CDS by a 2Aseparated translational unit representing the fluorescent transformation marker” in a more precise manner. In fact the 2A-flanked translational unit is a nonsense peptide, it doesn’t represent the fluorescent marker but arises from translation of the marker’s reverse-complement coding and regulatory sequences.

***Reply****: This was clarified. New text (underlined; removed text struck through): “ ~~Although~~ This was designed for read-though translation, where as well as Cas9, the ribosome would translate the reverse complement and coding sequences of the marker, followed by the zpg host gene product. However, our results suggest that this novel configuration did not allow for sufficient expression of ~~the~~ zpg ~~host gene product~~ from the zpgD allele in females.”*

The authors speculate that this design involving a 2A-flanked nonsense peptide could explain the reduction of ZPG protein expression. This could indeed be the case, but alternative explanations are possible. The presence of the Cas9 coding sequence itself may be sufficient to suppress zpg expression. Either way, this may be a translational decrease, or even alternative intron splicing involving the synthetic intron’s 5’ splice site in Cas9 and a 3’ splice junction from one of the downstream zpg introns.

***Reply****: This possibility is now mentioned, along with text suggested in a previous comment to clarify the contribution of the nonsense peptide to the zpg^D^ fitness cost. New text (underlined): “However, our results suggest that this novel configuration did not allow for sufficient expression of zpg from the zpgD allele in females. Alternatively, other transcriptional or translational mechanisms may have led to reduced zpg gene product (e.g. regulatory interference from the Cas9 coding sequence). Read-through translation and the resulting nonsense peptide arising from antisense 3xP3::mScarlet may have also contributed the observed fitness effects. Another method for avoiding gene-regulatory problems...”*

One thought to share with the authors, not necessarily to discuss in the paper: in risk assessment of the zpgD system (should it ever be used in the field), one aspect would be to assess whether natural malaria-susceptibility alleles could be genetically linked to zpgR. This would be undesirable as it would increase the frequency of malaria susceptibility genes hitch-hiking with zpgR ! This could be assessed in the lab by comparing vector susceptibility to P. falciparum of final, zpgR-rich populations to their initial, zpgS-rich population precursor. I agree that besides this caveat, the idea of replacing the Cas9 gene drive allele with a pre-existing natural resistance allele has great appeal.

***Reply****: We thank the reviewer for these considerations.*

First § of methods could be more straightforward. The meaning of “we used GFP as a vessel for a new fluorescence marker” is not very clear: if I understand correctly GFP sequence was simply substituted for a new fluorescence marker (if GFP sequence fragments are left over, they should be annotated in the Suppl. nucleotide sequence file I requested).

***Reply****: This was clarified, and annotations are available in the new genbank files. New text (underlined): “...we used the GFP as a vessel for a new bright, dominant, fluorescent marker, codon-optimised for A. gambiae (3xP3::AgScarlet). This new marker cassette contained intron donor and acceptor sites so it could be spliced from the GFP coding sequence, but was also re-coded to allow translational readthrough (and generation of a nonsense amino acid chain on the end of the GFP) in the event that splicing did not occur – see below).”*

Second § of Methods: worth mentioning whether the 2A-Scarlet cassette has been verified/recoded to be devoid of stop codons in frame with the Cas9-zpg coding sequences, referring to the new Suppl. sequence file.

***Reply****: Supplementary files are now referred to in the text.*

Eighth § of Methods: not sure I agree with “Note that because zpgD/zpgS females are not fertile (see below), all hemizygous offspring carried a zpgR allele in their genetic background.”
→ Can’t hemizygous offspring carry zpgD from a zpgD/zpgR mother and zpgS from a zpgD/zpgS father?

***Reply****: Due to homing being quite efficient in males (96%) most zpg^D^/zpg^S^ males will pass on zpg^D^, sometimes novel zpg^R^ alleles but when we sampled by Sanger we didn’t see unmodified zpg alleles making it out of the germline. We had already observed during the establishment and rearing of the strains that only the pairing with zpg^R^ alleles allowed us to obtain progeny from zpg^D^ females.*

Should homogenize usage of the terms –gote / gous throughout the text when used in the adjective position (hemizygote/hemizygous , homo- etc. but as a noun keep –gote)

***Reply****: This was fixed.*

A few typos : One “rapidly” too many on page 11 of the full pdf, hemiozygote in Methods, “to” is missing in “compared one effector alone”, it’s for its …effector in a wild population, singular/plural inconsistency in last sentence of acknowledgements.

***Reply****: This was fixed.*

**Reviewer** #3: This manuscript describes an important advancement to Integral Gene Drives, developed in mosquitoes previously by Nash et al. in 2019, in which the drive element (Cas9-expression) is integrated into an essential fertility gene in such a way as to incur a high fitness load and so be selected against, ensuring its depletion from a population overtime, while simultaneously effecting introgression of separate, non-drive, antipathogen transgenes into cage populations by the drive element. Specifically, the zpgD transgenic allele is shown to drive three different effector modules, while itself decreasing in frequency over generations. This is an important development in mosquitoes with multiple merits for the potential of confineable drives, but the greatest I see is in how useful it will be for testing multiple transgene arrangements, simultaneously at a larger scale. The focus on minimal design elements improves the potential fitness loads associated with any given transgene. These are of high importance to our field.

The authors provide a thorough genetic analysis of the activity of their transgenes at the molecular level, in genetic crosses and cage populations. Beside providing compelling evidence for their claims, they demonstrate that genetic/molecular phenomena must be carefully examined in genetic crosses and cage populations because different guideRNAs, effectors, insertion locations and expression strategies, can have very different effects.

The authors detail a thorough genetic analyses demonstrate technical molecular phenomena:

-Variation in the target site in zpgR individuals confers resistance to the drive which is demonstrated to be important to the drive dynamics. This set of experiments highlight the need for accurate documentation of alleles in the target population to predicting and understanding movement of the transgene through the population.

-guideRNAs very close together at the target drive the transgene differently 65% compared to ~95% in figure 2A, which is important in a drive.

-Readthrough translation is indeed inefficient in this system, which was useful to this design.

This manuscript is well written throughout. The visuals in the figure are effective at illustrating the complex genetics and the implications of the data. There is a nice review of effector genes so far available in the introduction.

Minor improvements to be made:

The terms “homing-induced homozygosity,” “gene-conversion” and others are useful in understanding what is happening with the drive in the germline, which isn’t intuitive to scientists outside the field, but the first term isn’t used until the discussion. Could you use some of this language earlier in the paper?

***Reply:*** *We have changed text in the results section to now read (new text underlined): “We also concluded that this homing was able to induce homozygosity of zpg^D^ in the germline leads to a loss of fertility in females, but not in males.”*

*In response to one of the editor’s comments (see their 2^nd^ additional comment), we have also added more background on the formation of resistance alleles within the results section. In this discussion various gene-drive terms and topics (e.g. gene-conversion) are introduced more thoroughly than previously.*

It is essential for reproducibility of these strategies and for application to other mosquito systems for annotated constructs (particularly, pZPGCas9Dr_v2_ZPG1 and pZPGCas9Dr_v2_ZPG2) to be made publicly available, such as on GenBank upon publication.

***Reply****: Annotated genbank files of these plasmids have now been made available as a supplementary files.*

Some improvements to the discussion should be made:

- The authors claim that there is an advantage in using a drive that is integrated in such a way as to be selected against. In this case integration of the Cas9- and marker-expressing cassette into the open reading from of zpg reduces the proper expression of this gene. Can the authors discuss existing data on drive elimination in a “neutral” location for perspective on this advantage?

***Reply****: We are not sure if we understand the comment correctly: Simply generating a fitness cost by interfering with zpg expression alone would not lead to the observed and modelled behaviour. It is the fact that homing in females causes dominant sterility that leads to rapid self-elimination. Because of this no combination of zpg^R^ and zpg^S^ allele frequencies in the population can lead to propagation of zpg^D^.*

*A Cas9 integrated at a neutral location or rather (if that is what is meant) driving by targeting a neutral site would also eventually disappear, assuming there is a certain cost for expressing the Cas9 protein (or associated marker). But it would first spread through the population and connected populations. This has been modelled for replacement drives but there aren’t many studies where the long-term behaviour of such drives (post-fixation) has been followed experimentally. Again, perhaps something else is implied by the reviewer’s comment.*

- None of the non-autonomous elements reached fixation in the cage population (or maybe 1 did-ScoG-AP2). With decreasing Cas9 in the population, would we expect to see fixation of any of the non-autonomous elements? Perhaps in many of the cages, by generation 6, the allele frequencies were high enough we might expect them to continue to increase, or would the begin to decrease from a fitness load?

***Reply****: The model suggests (for the starting frequencies of the 4 transgenes we have chosen) that fixation would occur only rarely/stochastically in these populations. Due to the rapid loss of zpg^D^ and hence Cas9 the non-autonomous payloads peak around an average transgene frequency of ~80-85% even in the best case where we assume that they themselves induce no fitness cost. This is reflected by data (cage data now provided) where the highest transgene frequency reached in any replicate population was 86% (generation 4, cage 3, AP locus).*

- With the reduction in egg laying demonstrated, does this strategy resemble a “reduce and replace” strategy?

***Reply****: This is an interesting point and a useful comparison to make for some aspects of zpg^D^’s potential behaviour in a wild release, relating somewhat to reviewer 1’s comment regarding migration. See also the response to reviewer 1.*

**Have all data underlying the figures and results presented in the manuscript been provided?**
Large-scale datasets should be made available via a public repository as described in the *PLOS Genetics* [data availability policy](https://eur01.safelinks.protection.outlook.com/?url=http%3A%2F%2Fjournals.plos.org%2Fplosgenetics%2Fs%2Fdata-availability&data=04|01|david-ellis@ucl.ac.uk|f129d2907f4245f4864c08da124cf2cc|1faf88fea9984c5b93c9210a11d9a5c2|0|0|637842419076066410|Unknown|TWFpbGZsb3d8eyJWIjoiMC4wLjAwMDAiLCJQIjoiV2luMzIiLCJBTiI6Ik1haWwiLCJXVCI6Mn0%3D|3000&sdata=jjTXHdF2Hbg1OrkVRkqSfRkp5A5iqRPlDDKXC53YSYc%3D&reserved=0), and numerical data that underlies graphs or summary statistics should be provided in spreadsheet form as supporting information.

Reviewer #1: **No:** I have some concerns about data management that I hope are easily fixable by the authors. I cannot find the raw data to accompany fertility assays and cage trials; I also cannot see a link to a data repository. Supplementary Table 1 appears to be infection data, the analysis of which I cannot see mentioned anywhere in the manuscript.

Reviewer #2: **No:** Nucleotide sequence of the plasmid knocked into the zpg locus

Reviewer #3: **No:** Annotated Construct Sequences should be deposited in GenBank or analogous resource

PLOS authors have the option to publish the peer review history of their article ([what does this mean?](https://eur01.safelinks.protection.outlook.com/?url=https%3A%2F%2Fjournals.plos.org%2Fplosgenetics%2Fs%2Feditorial-and-peer-review-process%23loc-peer-review-history&data=04|01|david-ellis@ucl.ac.uk|f129d2907f4245f4864c08da124cf2cc|1faf88fea9984c5b93c9210a11d9a5c2|0|0|637842419076066410|Unknown|TWFpbGZsb3d8eyJWIjoiMC4wLjAwMDAiLCJQIjoiV2luMzIiLCJBTiI6Ik1haWwiLCJXVCI6Mn0%3D|3000&sdata=0%2BMtYxnk%2BNtjx%2BLP7LbVq%2FA0W7crFfv83ZNcppisMcA%3D&reserved=0)). If published, this will include your full peer review and any attached files.


If you choose “no”, your identity will remain anonymous but your review may still be made public.


**Do you want your identity to be public for this peer review?** For information about this choice, including consent withdrawal, please see our [Privacy Policy](https://eur01.safelinks.protection.outlook.com/?url=https%3A%2F%2Fwww.plos.org%2Fprivacy-policy&data=04|01|david-ellis@ucl.ac.uk|f129d2907f4245f4864c08da124cf2cc|1faf88fea9984c5b93c9210a11d9a5c2|0|0|637842419076066410|Unknown|TWFpbGZsb3d8eyJWIjoiMC4wLjAwMDAiLCJQIjoiV2luMzIiLCJBTiI6Ik1haWwiLCJXVCI6Mn0%3D|3000&sdata=GfcQ9JjsgDIWeEp0yAgUO2p7bWlnlkfBI87V3T%2FJCq4%3D&reserved=0).

Reviewer #1: No

Reviewer #2: **Yes:** Eric Marois

Reviewer #3: **Yes:** Vanessa M. Macias
